# Causal Discovery Combining K2 with Brain Storm Optimization Algorithm

**DOI:** 10.3390/molecules23071729

**Published:** 2018-07-16

**Authors:** Yinghan Hong, Zhifeng Hao, Guizhen Mai, Han Huang, Arun Kumar Sangaiah

**Affiliations:** 1 School of Computer Science and Technology, Guangdong University of Technology, Guangzhou 510006, China; honyinghan@163.com (Y.H.); zfhao@gdut.edu.cn (Z.H.); 2 School of Physics and Electronic Engineering, Hanshan Normal University, Chaozhou 521041, China; 3 School of Mathematics and Big Data, Foshan University, Foshan 528000, China; 4 School of Software Engineering, South China University of Technology, Guangzhou 510006, China; hhan@scut.edu.cn; 5 School of Computing Science and Engineering, Vellore Institute of Technology, Vellore-632014, Tamil Nadu, India; sarunkumar@vit.ac.in or arunkumarsangaiah@gmail.com

**Keywords:** Bayesian causal model, causal direction learning, K2, brain storm optimization

## Abstract

Exploring and detecting the causal relations among variables have shown huge practical values in recent years, with numerous opportunities for scientific discovery, and have been commonly seen as the core of data science. Among all possible causal discovery methods, causal discovery based on a constraint approach could recover the causal structures from passive observational data in general cases, and had shown extensive prospects in numerous real world applications. However, when the graph was sufficiently large, it did not work well. To alleviate this problem, an improved causal structure learning algorithm named brain storm optimization (BSO), is presented in this paper, combining K2 with brain storm optimization (K2-BSO). Here BSO is used to search optimal topological order of nodes instead of graph space. This paper assumes that dataset is generated by conforming to a causal diagram in which each variable is generated from its parent based on a causal mechanism. We designed an elaborate distance function for clustering step in BSO according to the mechanism of K2. The graph space therefore was reduced to a smaller topological order space and the order space can be further reduced by an efficient clustering method. The experimental results on various real-world datasets showed our methods outperformed the traditional search and score methods and the state-of-the-art genetic algorithm-based methods.

## 1. Introduction

In recent years, the application of causal inference in bioinformatics has become more extensive, and plays a very important role in the development of this field. For instance, it is used for the discovery of the causal relationships between genes and the development of symptoms [1], and how to analyze the phenomenon of synthetic lethality [2,3] in biomedicine, which arises when a combination of mutations in two or more genes leads to cell death. Causal inference is different from the traditional statistical learning methods. The causal inference is the internal generative mechanism of the research data and the traditional statistical learning is the joint distribution of observation variables. The most significant difference between causality and relevance is whether or not to reflect the intrinsic relationship between the data. In scientific research, understanding the causal relationship of objects is crucial to predicting the laws of objects. Causal inference has already been applied in many fields, such as gene therapy, economic prediction, etc.

The problem of causal discovery or causal inference is generally formulated by a probabilistic graphical model where causal directions are represented by the directed edges [4]. In the causal inference algorithm, the techniques commonly used in local causality are conditional independent test (CI) method [5] and score & search method [4].

For example, Peter-Clack algorithms (PC algorithms) [5] determine causal relationships by finding out all the CIs in the given dataset, and the K2 algorithm [1] obtains the maximum score by searching for an optimal structure to discover causal relationships.

In general, a CI test method is used to detect the V-structure, and we can even infer the directed acyclic graph (DAG) [6] by the extension of the partial directed acyclic graph (PDAG). The accuracy of the above methods in causal inference is highly impacted by the number of the detected V-structures. In special cases, for example, without detecting the V-structure, the effect is poor. Therefore, the method cannot completely determine all edges and cannot distinguish the Markov equivalence classes, therefore often fails to uncover the true causal relationships contained in the given dataset if the number of equivalence classes is sufficiently large.

To distinguish causal direction in a non-experimental setting, some researchers recently resorted to using asymmetric relationships among variables under various hypothetical conditions. The additive noise model (ANM) proposed by Shimizu [7,8] is proved to be effective if the given data is generated by following linear non-Gaussian structural equation model. This method was later extended to nonlinear cases for continuous cases [9,10] as well as discrete cases [11,12].

Concretely, the existing ANM-based algorithms can be formulated as follows: assume there are two variables *x* and *y* satisfying a causal functional model *y* = *f*(*x*) + *ε*, where *f*(*) is an arbitrary square-integrable function and *ε* is an independent noise of *x*. If the joint distribution *P*(*x*,*y*) allows an ANM for one (forward) direction rather than the other one (backward), i.e., *x* cannot be obtained by a function of *y* plus an independent noise term, then the forward causal direction *x* → *y* is accepted as the true causal direction. The Post-Nonlinear (PNL) model [13] further extends ANM by making an additional function on the function *f*(*) such that *y* = *g*(*f*(*x*) + *ε*) with a bijective function *g*: *R* → *R*. More recently, some researchers have aimed to detect the asymmetry from an information-geometric perspective [14,15,16]. We can see that these methods assume that reversible and deterministic mappings can get the random variables independently. According to the previous works, these methods are used to examine the asymmetry causality by different techniques, and effect in the low dimension is very good, but poor in the nonlinear high dimensional causal inference between variables.

There are also some hybrid algorithms such as the hybrid algorithm (HYA) [1] and three phases causal discovery method (TPCDM) algorithm [17], to some extent, are able to find the causal relationships of multidimensional networks. The additive noise method (ANM) differentiates the parent nodes and the child nodes in the HYA algorithm and also detects the relationship between the neighbor sets and the sink nodes in the TPCDM algorithm. However, the experimental results show that the effect of the methods above are not very accurate, because it is difficult to detect a one-to-many network structure by ANM methods [10,18,19,20,21,22,23,24,25,26,27].

We can see that all these methods for learning causal structure are unreliable, or the time complexity is so very high that we often cannot get the result in an acceptable time. In this situation, we resort to optimization algorithms.

Then, we study the optimization algorithms. Problems existing in many real worlds can be classified as optimization problems. The traditional optimization algorithm is based on a single point, such as gradient descent algorithm, which is a point that moves in the opposite direction of the gradient function. The traditional optimization algorithm mainly solves the problem of a single peak; it is easy to obtain the local optimal solution in the case of complex multiple modes and nonlinear problems.

In recent years, the swarm intelligence (SI) algorithm has been a topical research topic in solving the problem of multiple peaks. Swarm intelligence algorithms are used to solve problems by learning some life phenomena or natural phenomena in nature, which includes the characteristics of self-organization, self-learning and adaptability of natural life phenomena. Especially in 2011, a new SI algorithm [28] called “Brain Storm Optimization” (also known as Brainstorm optimization, BSO) was proposed, which was inspired by human brainstorming activities. The paper demonstrates the ability of BSO to solve optimization problems by testing two basic functions. Based on the idea of human creative problem-solving, a new swarm intelligence algorithm, Shi’s [9] optimization algorithm, was proposed. Unlike traditional swarm intelligence algorithms, such as ant colony optimization (ACO) and artificial bee colony (ABC), the BSO algorithm is the first one to solve the problem based on human creative thinking. Humans are the smartest animals in the world, and the BSO algorithm, which is inspired by their social behavior, is considered a promising method [9]. Shi [9,10] elaborated the thought and implementation process of BSO algorithm, and used the classical test function to simulate the BSO algorithm, and the results showed the effectiveness of BSO algorithm. However, there is still a problem of precocious maturity, and it is necessary to further study to optimize the algorithm itself, improve the effect of BSO algorithm [11].

In this study, we design an efficient method to support causal discovery by combining K2 with Brain Storm Optimization Algorithm (K2-BSO). We use the score returned by the K2 algorithm as the fitness function, and design an elaborate distance function for the clustering step in the BSO according to the mechanisms of K2. The graph space therefore was reduced to a smaller topological order space and the order space can be further reduced by an efficient clustering method. After a optimal causal order is returned by BSO, we run K2 to search for the optimal causal structure, and output the causal skeleton. In the case of high dimensions, the following methods are first used to process the skeleton. We split the causal skeleton into *n* (the number of variables in the skeleton) smaller sub-skeleton, and employ ANM to detect the causal directions between the target variables and all its parents from each causal skeleton. Consequently, we obtain a partial DAG (PDAG) w.r.t. each sub-skeleton. By merging all the PDAGs, the whole structure corresponding to the high dimensional causal network w.r.t. the given dataset is finally reconstructed. K2-BSO is designed for a certain problem, and the most different thing from other BSO methods should be the clustering procedure, since in the our design, we need to measure the distance between two node sequences in term of the corresponding orders instead of two sequences perset, therefore the existing clustering methodologies used in other BSO methods like those mentioned in [29,30,31] are not applicable for our case.

The rest of this paper is organized as follows: Section 2 briefly summarizes these definitions. Then we focus on the introduction to the basic concepts, algorithm flow and advantages and disadvantages of K2 and BSO algorithms in Section 3. The details of Causal Discovery combining K2 with Brain Storm Optimization Algorithm are discussed in Section 4. The correctness and performance characteristics of three algorithms are shown in the Section 5. Section 6 gives detailed experimental results. Finally, the conclusions are drawn in Section 7.

## 2. Definitions

In this section, we will introduce several basic definitions applied in our method. The concepts of the D-separation, V-structure and Additive noise model, which is described as follows:

A causal network can be expressed as a directed acyclic graph (DAG) *G_N_* = {*V_N_,E_N_*}, in which *E_N_* = {*e*_1_,*e*_2_,…,*e_n_*} and *V_N_* = {*x*_1_,*x*_2_,…,*x_n_*} denote the sets of edges and nodes in *G_N_*.

***A. D-separation***

**Definition** **1.** *(d-Separation). Assume L is a path from x_i_ to x_j_, and is blocked by a set of variables Z if one of the following conditions holds:*

(1) L either contains a chain, *x_i_* ← *x_k_* ← *x_j_*, and *x_k_* ∈ *Z*,
(2) or a fork, *x_i_* ← *x_k_* → *x_j_*, and *x_k_* ∈ *Z*,
(3) or a collider, *x_i_* → *x_k_* ← *x_j_*, and *x_k_* ∉ *Z*, and no descendent of *x_k_* is contained in *Z*.

We say a set *Z* separates two disjointed sets *X_i_* and *X_j_* (*X_i_*, *X_j_* ⊆ *V_D_*) if *Z* blocks each path between *X_i_* and *X_j._*

***B. V-structure***

**Figure 1:**
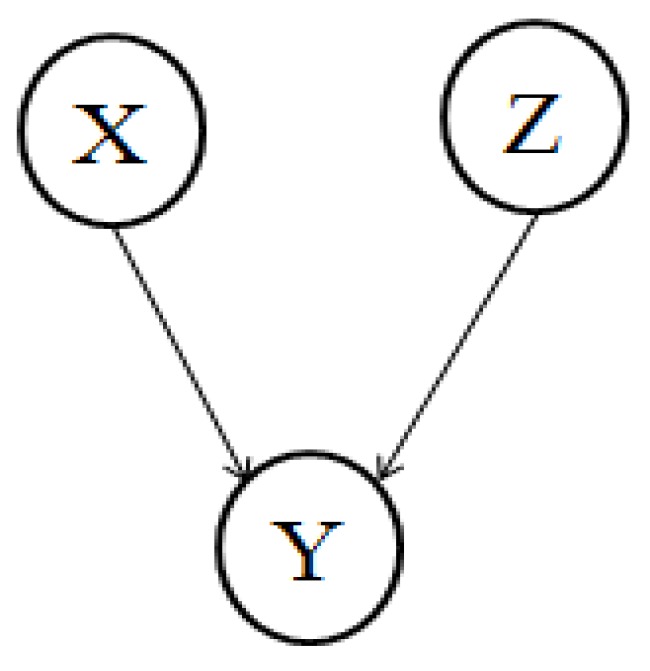
Illustration of a V-structure.

**Definition** **2.** *(V-Structure). Given three variables x, y, and z. If x and z are the parent nodes of y, and no other edge is existing between x and z, then x, z and y together form a V-structure. As shown in Figure 1.*

***C. Additive noise model***

**Definition** **3.** *(Additive noise model (ANM for short)) ANM is represented by a collection of n equations S = *{*S_*1*_*,*S_*2*_*,…*S_n_*}*: S_i_:x_i_ = f_i_*(**$x_{pa_{\left(i\right)}}$) + *ε_i_*, *i* = {1,2,…,*n*}, *where* $x_{pa_{\left(i\right)}}$ *is the direct parent set of x_i_, the noise terms ε_i_ are jointly independent, and are independent from x_i_.*

It can be seen that the data-generating processes of *X* can be expressed as:

$$S_{i}:\;x_{i}=\varepsilon_{i},i=\left\{1,\;2,\;\ldots ,\;k\right\}\;(\mathrm{the}\;\mathrm{root}\;\mathrm{nodes})$$

$$S_{j}:\;x_{j}=f_{j}\left(x_{pa_{\left(j\right)}}\right)+\varepsilon_{j},\;j=\left\{1,\;2,\;\ldots ,\;n-k\right\}\;(\mathrm{the}\;\mathrm{other}\;\mathrm{nodes})$$

As shown aforementioned ANM provides a way for finding casualties by using the assumption of additional noise data generation process rather than satisfying Markov conditions.

## 3. The K2 and Brain Storm Optimization

In this section, we first introduce the K2 algorithm. Then, the basic concepts, algorithm flow and advantages and disadvantages of BSO algorithm are introduced in detail. All in all, the whole process of the K2 and Brain Storm Optimization can be described as follows.

### 3.1. The K2 Algorithm

K2 Algorithm, developed by Cooper and Herskovits in 1992, is a Bayesian Network Structure learning algorithm based on the score search method. It is a classical algorithm in the Bayesian Network Structure field with excellent learning performance [32].

As we all know, Bayesian Network Structure Learning aims to find the Bayesian Network Structure *B_S_* which best connects with *D* through the analysis of data set *D*. That is the Bayesian Network Structure *B_S_* with maximum posterior probability *P*(*B_S_*|*D*). Because *P*(*B_S_*|*D*) = *P*(*B_S_*|*D*)/*P*(*D*) in which *P*(*D*) is a constant, what we find is the network structure *B_S_* that maximizes the probability *P*(*B_S_*|*D*), that is:

(1) $$\max\left[P\left(B_{S},D\right)\right]=c\prod_{i=1}^{n}\max\left[\prod_{j=1}^{q_{1}}\frac{\left(r_{i}-1\right)!}{\left(N_{ij}+r_{i}-1\right)!}\prod_{k=1}^{r_{i}}N_{ijk}!\right],$$

where *c* is the a priori probability *P*(*B_S_*|*D*) of each network structure, which is meant to be a constant because in the algorithm of K2, it is assumed that every network structure *B_S_* has the same probability; *n* is the number of nodes; *r_i_* is the number of values of node *X_i_*; *π_i_* is parent nodes set of node *X_i_*; *q_i_* is the number of configurations of *π_i_*; *N_ijk_* is the sample number of node *X_i_*, which takes the value of *k*, and its parent set is the *j*th configuration in data set *D*; $N_{ij}=\sum_{k=1}^{r_{i}}N_{ijk}$.

As is showing above, K2 Algorithm uses Equation (1) as a score function to learn the Bayesian Network Structure. From Equation (1), the score function can be decomposed, that is, it can be seen as products of *n* local structures, which is made up of each node *X_i_*, i=1,2,…,n and its corresponding parent nodes set. Then the following equation is derived:

(2) $$g\left(X_{i},\pi_{i}\right)=\sum_{j=1}^{q_{i}}\frac{\left(r_{i}-1\right)!}{\left(N_{ij}+r_{i}-1\right)!}\prod_{k=1}^{r_{i}}N_{ijk}!$$

(3) $$G\left(B_{S},D\right)=\sum_{i=1}^{n}g\left(X_{i},\pi_{i}\right)$$

so we can maximize *G*(*B_s_,D*) if we maximize every local structure’s scores *g*(*X_i_*,*π_i_*), inevitably also maximizing the scores of the whole Bayesian Network Structure (Equation (1)). According to this idea, given nodes order *ρ* and the upper limits *μ* of each node’s parent nodes, the K2 algorithm can use Greedy Searching to find each node’s parent nodes in turn so as to finally construct a whole complete Bayesian Network. The concrete method is as follows: firstly, for every node *X_i_*, *i* = 1,2,…,*n*, constantly choose the former nodes in former nodes’ set from nodes order into parent set *π_i_* of node *X_i_*, making the score function *g*(*X_i_*,*π_i_*) of *π_i_* and *X_i_* continuously increase. The above process cannot stop until function *g*(*X_i_*,*π_i_*) does not increase any more when adding nodes. In that process, we need to limit that the parent node’s number should be under *μ*.

As is known to all that the K2 algorithm has two prerequisites, given nodes order *ρ* and the upper limits *μ* of each node’s parent nodes. With these two prerequisites, it can obtain a very good learning performance, but in most situations, we can’t always meet the above prerequisites, causing difficulties in the application of the K2 algorithm.

### 3.2. Brain Storm Optimization

#### 3.2.1. Brainstorming Algorithm Principle

Inspired by human behavior patterns, in 2011, a human brainstorming process was proposed for the first time by Shi et al., called Brainstorming Optimization Algorithm (BSO). Shi’s article expounds the thought and realization process of BSO in detail, and simulates the BSO algorithm with classical test function, and the experimental results show that the BSO algorithm is effective. However, there are some deficiencies in the new algorithm, such as easily falling into local optima, resulting in premature convergence. Therefore, it is necessary to improve the BSO algorithm and optimize the algorithm so as to improve its effect [33,34,35,36,37,38].

The concept and theory of the basic BSO algorithm is derived from the simulation of the human brainstorming process. A brainstorming meeting needs a moderator, a number of owners to solve problems, and a group of parliamentarians with different backgrounds. Since parliamentarians have different backgrounds, different experiences and different ways of thinking, one problem will get different solutions. The moderator, in accordance with the four Rules of the Conference (see Table 1), presides over the meeting and gets solutions from as many as possible [38,39,40,41,42,43]. The algorithm needs a skilled host, with no or almost no problem-solving knowledge, so as not to lead host into bias, and also the host cannot engage in new ideas until all ideas are proposed. The host can divide it into K classes, and for each class, people can diversify their thinking and propose better solutions until they get the best solution. The BSO algorithm gets its inspiration from this model and then simulates the process. In the BSO algorithm, the feasible solution of each optimization problem is a quantity of information in the search space, all the information has an adaptive value which is determined by the function of optimization, and then the optimal information is iterated by clustering and learning all kinds of excellent information.

The brainstorming session procedure is as follows:

(A) Assemble as many parliamentarians with different backgrounds as possible;
(B) Get the solutions based on the brainstorming rules in Table 1;
(C) Choose a scheme as the best solution for the current problem from each of the problem-solving owners;
(D) Generate new schemes from the schemes selected in C according to the rules in Table 1
(E) Choose a solution from the idea of each problem-solving owner in D as the best solution for the current problem
(F) Randomly select a scheme as a clue to generate new schemes in the case of meeting the Rules in Table 1;
(G) Each problem-solving owner chooses a scheme from F to be the best solution for the current problem;
(H) Get the best solution that is desired by considering merging these programs.

#### 3.2.2. BSO Algorithm Steps

The brainstorming algorithm is mainly composed of two modules: a clustering module and a learning module. In the clustering module, the algorithm uses the clustering method to gather the information into K classes, and the cluster center in each class is the optimal value. The algorithm is optimized by learning, also the information in each class is in parallel. Similarly the local search is promoted, and the algorithm jumps out of the local optimization through the cooperation between classes and the mutation operation, which promotes the global search. The convergence of the algorithm is ensured by the optimization process of cluster center, and the process of optimizing the information variation in the class ensures the diversity of the algorithm population. Each individual in the BSO algorithm represents a potential problem solution that is updated by the individual’s evolution and fusion, a process similar to that of the human brainstorming process [44,45,46].

The implementation of BSO algorithm is simpler:

(1) Obtain the solution of *n* potential problems, then divide *n* individuals into M class by K-means method, the individual in each class is sorted by evaluating these *n* individuals, and the optimal individual is selected as the central point of the class;
(2) Randomly select the central individual of a class and determine whether it is replaced by a randomly generated individual according to the probability;
(3) to update the individual, the way is updated by the following four ways: (a) randomly select a class (the probability of selection is proportional to the number of individuals within the class), the random perturbation is added to the class center to produce a new individual; (b) randomly select a class (the probability of selection is proportional to the number of individuals within the class) and randomly select an individual in the selected class, plus a random perturbation to produce a new individual; (c) randomly selected two classes, the fusion of the class center and the random perturbation to produce a new individual; (d) randomly select two classes, randomly select an individual in each class, and then add a random perturbation to create a new individual. By adjusting the parameters to control the proportion of the above four ways to produce new individuals. After the new individual generation, compared with the original individual, the final selection of the best one to retain to a new generation of individuals, repeat the above operation, the *n* individual to update each one, produced a new generation of *n* individuals.

This loops until the upper limit of the preset individual update algebra is reached. In the third step, the update of the individual has four ways to produce a new individual process; the selected amount of information plus a Gaussian random is worth the new amount of information, such as the following Equation (4):

(4) $$X_{new}^{d}=X_{selected}^{d}+\varepsilon \times n\left(\mu ,\sigma \right),$$

where $X_{new}^{d}$ is the *d* dimension of the new information, $X_{selected}^{d}$ is the *d* dimension of the selected information, n(μ,σ) is the Gaussian function whose mean value is *μ* and variance is *σ*; *ε* is a weight coefficient which is described by Equation (5):

(5) ξ=logsig((0.5×max_iteration−current_iteration)/k)×rand(),

where log*sig*() is a s-type logarithmic transfer function, and *max_iteration* is the maximum number of iterations, while *current_iteration* means the current number of iterations; *k* can change the slope of the function log*sig*(), *rand*() is the random value between (0,1).

## 4. The K2-BSO Method

In this section, the details of the K2-BSO method are given, we show that this method is able to discover causation combining K2 with the Brain Storm Optimization algorithm. All in all, the whole process of causality is deduced, which is described as follows:

### 4.1. Skeleton Learning Phase Based on K2-BSO

The Additive Noise Model (ANM) could find out the causal relationships correctly between variables in sparse causal networks, but this model would encounter multiple challenges when applied to high-dimensional complex network structures [12]. First of all, high-dimensional causal networks contain a large number of variables, and the causal relationships between them are very complex, so the algorithm requires the ability to quickly search. Causal relationship references based on the traversal method will face all possible network structures, which leads directly to the insufferable computational complexity, the storage space overflow and other problems. The K2 algorithm needs to satisfy two prerequisites, given nodes order and the upper limits of each node’s parent nodes. However, it is difficult to make it in fact. What’s more, the K2 algorithm is easy to fall into the local optimal solutions while the BSO algorithm could get rid of local optimizations. Therefore, the combination of the algorithm K2 and BSO can effectively solve the structural learning problem of causal network structure. As discussed in the previous section, there are three points we need to note:

(1) What needs to be optimized is the causal order that will highly affect the accuracy of K2. Generally, an input order approaching the actual topological order of the underling causal network will return the highest score and most similar causal structure.
(2) The fitness function is easy to be chosen, that is the score return by K2.
(2) The clustering method of BSO should be redesigned; all the distance function likes [46] cannot be directly applied to this case, as what we consider is the topological order. We design a new distance function like this:

Step I. Given two orders $R_{1}$ and $R_{2}$, for each variable in $R_{1}$, we find the same variable in $R_{2}$, assume it is $v_{1}$.

Step II. Consider *n* variables in front of $v_{1}$ in $R_{1}$, and m variables in front of $v_{1}$ in $R_{2}$, we calculate the number of the repeated variables in n+m variables.

Step III. By literately sum up the repeated variables w.r.t. every variable in $R_{1}$ (or $R_{2}$), we get a number, and let this number as the distance between $R_{1}$ and $R_{2}$.

We note that, the clustering step is crucial to the BSO, as shown before, our distance function is designed based on the mechanism of K2, which will highly improve the clustering performance in BSO.

### 4.2. Direction Learning Phase

Algorithm 1 can obtain the skeleton of network returned by K2-BSO. Because the K2 can only examine a set of Markov equivalence classes rather than the realistic causal structure, we aim to detect the remaining directions of the output skeleton for distinguishing this equivalence in this section. Because of the existence of Markov equivalence classes, the structural learning methods are generally difficult to infer all causal direction. On the other hand, the ANM provides an effective way to learn causal direction in low-dimensional cases. Note that, we get the causal skeleton, then we can separate the causal skeleton S into *n* sub-skeletons (*S_i_*,…,*S_n_*) which contain a target node *X_i_* and all its neighbor nodes *N_i_* according to S. In general, these sub-skeletons are generally low-dimensional and therefore can be solved by using ANM. The way to orient the edges of a skeleton in ANM method is described as follows:
**Algorithm 1.** Skeleton learning based on K2-BSO.**Input:** dataset *X*, population size |V|. **Output:** the skeleton w.r.t. *X*. 1: Randomly generate *n* potential causal order $R=R_{1}∼R_{n}$; 2: Cluster *R* into m clusters $C=C_{1}\sim C_{m}$; 3: **For** each $R_{i}$ Score_i_ = K2($R_{i}$); 4: **End For** 5: Score = Score_1_ ~ Score_n_; 6: R_optimal_ = BSO (*X*, *R*, Score, *C*); 7: G_optimal_ = K2(R_optimal_); 8: X = G_optimal_; 9: **return** the causal skeleton *X*.


Firstly, consider a given dataset *X* = {*X*_1_,*X*_2_,…,*X_n_*} with index *V* = {1,2,…,*n*}. *X* corresponds to an *n*-dimensional DAG *G* = {*V*,*E*}, where *E* represents the edges of *V*. Assume that *X* is generated by the following way: each variable *X_i_* ∈ *X* corresponds to one node *i* ∈ *V* in G, and is determined by a causal function $X_{i}=f_{i}\left(x_{pa_{\left(i\right)}}\right)+\varepsilon_{i}$ in which *f_i_* is nonlinear, $x_{pa_{\left(i\right)}}$ is the parent of *x_i_*. The noise terms *ε_i_* have a non-Gaussian distribution and are jointly independent.

In the issue of seeking out the causal direction, we aim to seek out all the parent nodes (contained in *N_i_*) amount to each target *X_i_* from *S_i_*. On the basis of the mechanism of ANM, we denote the homologous remains between *X_i_* and each candidate parent set *C_ik_* as *X_i_* = *f*(*C_ik_*) + *ε_i_* by using GPR, and we test whether *C_ik_* and *ε* are statistically independent. If they are independent we accept the model *C_ik_* → *X_i_*; if not, we deny it. In this phase, we measure the independence by using the kernel-based conditional independence (KCI) test. The details of causal directions inference from a output causal skeleton is presented in Algorithm 2.
**Algorithm 2.** Learning causal direction from a sub-skeleton.**Input:** sub-skeleton $S_{i}$ and the corresponding target node $X_{i}$ with all its neighbors $N_{i}$. **Output:** the direction between $X_{i}$ and (partial) $N_{i}$. 1: **For** each candidate parent set $C_{ik}$; 2: fit $X_{i}$ and $C_{ik}$ to ANM; 3: **if**
ε is independent of $C_{ik}$
**then** 4: accept $C_{ik}$
→
$X_{i}$; 5: **end if** 6: **end for**


### 4.3. K2-BSO Framework (Algorithm 3)

We first present the details of the K2-BSO method:

Step 1. Learning the causal skeleton S by algorithm 1.
Step 2. Split *S* into *n* sub-skeleton $S_{1},\ldots {,S}_{n}$ according to each node *X_i_* contained in *S*.
Step 3. Perform Algorithm 2 for each sub-skeleton *S*_i_.
Step 4. Merge all the partial results and output the final causal structure.

**Algorithm 3.** K2-BSO framework.**Input:** Dataset *X*, threshold *k* **Output:** Causal structure *G*. 1. Set Dimension *X* to *n*; 2. **if** (*n* < *k*) **then** 3. S=Algorithm 1(X); G=Algorithm 2(X, S); 4. **else** 5. Split S into *n* sub-skeleton $S_{1},\ldots {,S}_{n}$ according to each node $X_{i}$ contained in *S*; 6. **For** each $S_{i}$ in *S* 7. $S_{i}=Algorithm\;1\left(X_{i}\right)$; $PDAG_{i}=Algorithm\;2\left(X_{i},\;S_{i}\right)$; 8. Merge all $PDAG_{i}$ to *G*; 9. **End for** 10. **end if** 11. **return** the final causal structure *G.*


## 5. The Correctness and Performance of the Algorithms

In this part, we analyze theoretically about the respective characteristics of the correctness and performance with the three algorithms (K2-Random, K2-BSO, K2-GA).

First, we discuss the K2-Random algorithm. It is a traditional method, and there is not much optimization process. The main process is: first step, randomly obtain *p* data sort, then sort the score from the top to the bottom and select the highest score. The second step is to continue to randomly obtain *p* data sort, found the highest score Tscore, until 10 consecutive times are the same highest score, and end the program; this method is very easy to enter the local optimization state, but the experimental result is unstable.

Second, we discuss the K2-BSO algorithm, which is the method proposed in this paper. It is better to avoid local optimization problems. The main process is: first step, randomly obtain *p* data sort, then sort the score from the top to the bottom and obtained *m* data sort by clustering method. The second step is to obtain *m* new subclasses by random perturbation about *m* subclasses by the BSO algorithm. Then we reevaluate the score until the score converges.

Third, we discuss the K2-GA algorithm. The main process is: The main process is: first step, randomly obtain *p* data sort, Then sort the score from the top to the bottom and select the highest score until the score converges. The second step is to obtain *p* new data sort by means of Genetic Algorithm (GA) method with randomly perturbation the highest ranking data. Then sort the score from the top to the bottom to obtain the highest score Tscore.

In summary, the first algorithm in time complexity is the best, but the accuracy rate is the lowest and unstable; the second algorithm and the third algorithm’s time complexity are the same, especially with the increase of network dimensions, second algorithms tend to advance convergence faster than the third algorithms, and the accuracy of the second algorithms is better than the third algorithm. Next, we’ll use real data to validate three algorithms in the next chapter.

## 6. Experiments

In this section, we evaluate our proposal on eight real-world datasets that cover a variety of applications including Small Networks (Asia, Sachs), Medium Networks (Child, Alarm), Large Networks (Barley, Win95pts), and Very Large Networks (Pigs, MINUN) that cover a variety of applications, including, medicine (ASIA, SACHS, CHILD and ALARM), agricultural industry (BARLEY), system troubleshooting (WIN95PTS) and bioinformatics (PIGS and MUMIN) are available at “http://archive.ics.uci.edu/ml/datasets.html”. The structural statistics of the eight networks are summarized in Table 2.

In this group of experiments, our proposed method is compared with other two mainstream causal discovery methods—K2-Random (Causal Discovery combining K2 with Random) method and K2-GA (Causal Discovery combining K2 with Genetic Algorithm) method. We evaluate these methods by different sample size at 250, 500, 1000, 2000, respectively. We use three criteria, Recall, Precision, and F1 to evaluate these methods, which are defined as follows:

(9) Recall=(Inferred directions∩Actual directions)/(Actual directions),

(10) Precision=(Inferred directions∩Actual directions)/(Inferred directions),

(11) F1=(2×Recall×Precision)/(Recall+Precision),

Obviously Precision is the actual fraction of inferred causality with respect to a true graph. Similarly, Recall is the part of actual causality found by the algorithm. F1 is the organic combination of Precision and Recall which can serve as the accuracy standard for our algorithms.

The experimental environment is as follows:

(1) CPU of the physical host: CPU E5-2640 v3, 2.60 GHz (2-way 8-core);
(2) Platform belongs to the cloud platform version from Bingo Cloud: v6.2.4.161205143;
(3) Memory is 24 G.

**Figure 2:**
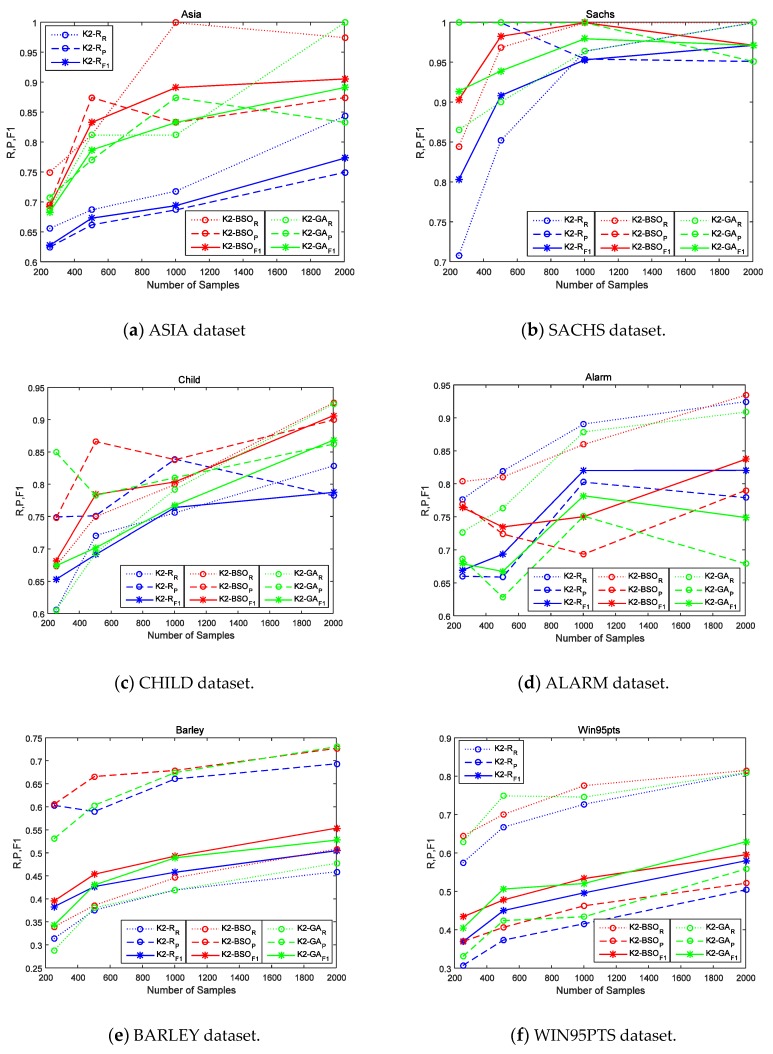

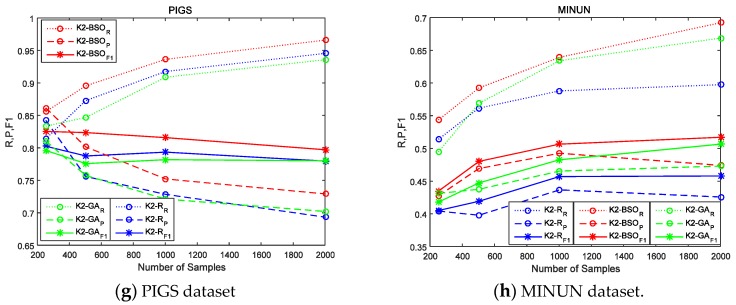
R, p & F1 of the K2-R, K2-BSO and K2-GA with eight dataset: (**a**) ASIA dataset; (**b**) SACHS dataset; (**c**) CHILD dataset; (**d**) ALARM dataset; (**e**) BARLEY dataset; (**f**) Win95pt dataset; (**g**) PIGS dataset; (**h**) MINUN dataset.

As shown in Table 3, we can see that the K2-Random runs much faster than the other two algorithms. However, as showed in Figure 2, the accuracy of K2-Random is lowest, this means K2-Random easily falls into a local optimum. One can imagine that if we use K2-Random to test all possible causal orders detailed we can obtain the best score, but we usually cannot get the final result in an acceptable time, because the time complexity of such an exhaustive algorithm reaches the upper limit.

On the other hand, we can see that in the small networks, K2-GA runs faster than K2-BSO. However, as the size of the networks grows, the running time of K2-BSO increases slower than that consumed by K2-BSO, and the running time of the two methods tend to be very close. We can see that in the case of WIN95PTS, K2-BSO runs much faster than K2-GA. What is the most different between K2-BSO and K2-GA in the task is that K2-BSO performs a clustering step, which can greatly reduce the convergence time. Recall that, the clustering step in K2-BSO also costs time. Therefore, when the causal network spends more time in clustering step, K2-BSO is probably slower than K2-GA, while for a network to spend less time in the clustering step, theoretically K2-BSO runs much faster than K2-GA. Accordingly, the specific structure of a certain causal network weighs heavily on total time.

As shown in Figure 2, K2-BSO achieves the better score in the majority of cases, which means that the clustering step can not only improve the convergence speed on the basis of the number of iterations, but also prevent K2-BSO from falling into local optima. Even the largest network PIGS shows that the F1 score is 2% better than K2-GA.

Figure 2 also shows the main trends of the indexes (Recall (R), Precision (P), and F1) of the three algorithms (K2-R, K2-BSO, K2-GA), with different samples [250,500,1000,2000] in eight datasets, including ASIA, ALARM, SACHS, BARLEY, CHILD, Win95pt, PIGS and MINUN. The blue line ‘o:’ represents the numerical trend of the Recall of K2-Random; the blue line ‘o--’ indicates the numerical trend of the Precision of K2- Random; the blue line ‘*—’ indicates the numerical trend of the F1 of K2-Random; The red line ‘o:’ represents the numerical trend of the Recall of K2-BSO; the red line ‘o--’ indicates the numerical trend of Precision of K2-BSO; the red line ‘*—’ indicates the numerical trend of F1 of K2-BSO; The green line ‘o:’ represents numerical trend of the Recall of K2-GA; the green line “o--” indicates the numerical trend of the Precision of K2-GA, and the blue line “*—” indicates the numerical trend of the F1 of K2-GA.

Figure 2a shows the curves of the three methods (K2-R, K2-BSO, K2-GA) with different samples in the data set ASIA. It can be seen that the red curve basically goes above the green one and the blue one, which means that K2-BSO’s indexes R, P, F1 are higher than that of K2-R and K2-GA, thus proves that K2-BSO algorithm is better than K2-R algorithm and K2-GA algorithm.

Figure 2b–e show the curves of the three algorithms (K2-R, K2-BSO, K2-GA) with different samples in data sets SACHS, CHILD, ALARM and BARLEY. It can be observed that the results are similar to that in Figure 2a, that K2- BSO’s indexes R,P,F1 are higher than that of K2-R and K2- GA, thus also proves that K2-BSO algorithm is better than K2-R algorithm and K2-GA algorithm. Figure 2f–g shows the curves of the three methods (K2-R, K2-BSO, K2-GA) with different samples in data set WIN95PTS, PIGS and MINUN. WIN95PTS is a 76-dimensional network, PIGS is a 441-dimensional network and MINUN is a 1041-dimensional network, so they belongs to the high dimensional networks. We can see from Figure 2f that the curve of the blue value is the lowest; with sample 500 and 2000, the value of the green curve is slightly higher than that of the red curve, while on the whole, the red curve goes above the green. However, when we refer to Table 3, it is obvious that the execution time of K2-BSO is much less than that of K2-GA, which means the K2-BSO is better than the other algorithms in this network. On the other hand, Figure 2g shows that the curves are slightly different from the form’s results, the Recall of the three methods grows with the increase of sample size while the Precision reduces with the increase of sample size.

The reason for such a difference is that PIGS is a genetic network, that is, PIGS has a very complex structure where some nodes connect with many neighboring nodes, for example, the maximum degree is 41 (maximum in-degree is 2). Accordingly it is difficult for the subroutine K2 to remove these in-direct causal relationships. Even so, it can be seen that the F1 score of K2-BSO is still 2% better than those of K2-R and K2-GA. Figure 2h shows that even in the case of MINUN network of more than 1000 dimensionality, K2-BSO works much better than K2-GA and K2-R. These results demonstrate that our method K2-BSO is much reliable than the counterparts in more complexity and higher-dimensional cases, and also shows that K2-BSO is able to learn the causal structure from a dataset with hundreds of variables. In summary, K2-BSO performs better than K2-GA if the accuracy and execution time are combined, so in our future work, we will continue to perfect the K2-BSO algorithm, making it adapt to high-dimensional network accuracy problems at the cost of some appropriate execution time.

## 7. Conclusions

To reduce the search space of graphs is important in causal relationship discovery; however, the existing methods show inefficiency for large scale causal networks. In this work, an improved causal structure learning algorithm combining K2 with brain storm optimization (BSO) called K2-BSO is presented to alleviate this problem. In contrast to other evolutionary algorithms based on the search and score methods, K2-BSO has two significant advantages, (1) K2-BSO searches optimal topological order of nodes instead of graph space. The order space should be much smaller than the whole graph space. In this phase, an elaborate distance function is introduced for clustering nodes’ orders in BSO based on the mechanism of K2. The graph space therefore is reduced to a smaller topological order space that can be further reduced by an efficient clustering method. (2) Our method is designed through the following split and merge strategy, the original dataset is split into a set of subdata sets in the first place. The BSO will run on these subdata sets to recover the corresponding substructures. Here we further use additive noise model approach to rectify the direction of the erroneous orientation or the side without direction. We eventually merge all these substructures and obtain the entire structure of the graph. The experimental results on various causal networks showed our method could outperform the traditional search and score method and the state-of-the-art genetic algorithm-based method.

## Figures and Tables

**Table 1:** Osborn’s Original Rules for Idea Generation in a Brainstorming Process.

Rule 1 No bad ideas, every thought is good
Rule 2 Every thought has to be shared and recorded
Rule 3 Most ideas are based on existing ideas, and some ideas can and should be raised to generate new ideas
Rule 4 Try to produce more ideas

**Table 2:** Statistics on the network.

Network	Nodes	Edges	Avg Degree	Maximum in-Degree
ASIA	8	8	2	2
SACHS	11	17	3.09	3
CHILD	20	25	1.25	2
ALARM	37	46	2.49	4
BARLEY	48	84	3.5	4
WIN95PTS	76	112	2.95	7
PIGS	441	592	2.68	2
MUMIN	1041	1397	2.68	3

**Table 3:** Comparisons between three algorithms on execution time.

Dataset	Sample	K2-Random			K2-BSO			K2-GA		
		Best	Mean	Worst	Best	Mean	Worst	Best	Mean	Worst
ASIA	250	1.2555	1.749	2.6157	3.0185	4.5585	5.8543	2.8367	4.2504	5.5427
ASIA	500	1.085	1.4656	1.9224	3.2043	4.3753	6.3392	2.1125	4.8925	6.4698
ASIA	1000	1.5104	1.7994	2.2099	4.7037	5.0472	5.3491	3.1055	5.1146	8.2191
ASIA	2000	2.2676	2.4993	2.6629	3.3815	3.9774	4.3823	4.1878	5.506	8.1496
SACHS	250	4.5248	5.025	5.3196	6.2266	6.4963	6.6793	3.02	4.6432	7.501
SACHS	500	2.4084	3.6746	5.2433	8.7819	11.2107	15.6879	5.5198	8.1696	8.6039
SACHS	1000	2.7062	3.5759	4.0197	8.3591	11.7962	15.3562	5.4356	8.6128	12.8977
SACHS	2000	3.4966	4.6789	6.4649	9.3677	10.7807	11.5173	6.1085	6.1516	6.1762
CHILD	250	7.7092	9.117	12.5631	28.0133	29.961	31.325	21.1455	25.986	30.974
CHILD	500	8.9414	11.8924	14.1062	17.7665	28.9292	41.265	30.2484	34.1696	38.9523
CHILD	1000	10.059	17.5069	28.2669	33.4957	38.4505	43.9597	23.6723	24.608	25.3404
CHILD	2000	10.709	13.8898	18.3508	30.1317	58.6901	78.3929	21.3343	38.465	47.0478
ALARM	250	46.647	71.9888	86.3291	217.3178	266.3269	355.1756	147.9969	283.8744	371.7365
ALARM	500	94.125	103.2041	119.5914	227.3739	293.7428	377.54	133.6973	388.0371	519.749
ALARM	1000	62.140	92.5241	125.1668	157.303	247.1288	294.8618	144.2063	316.8503	475.2459
ALARM	2000	95.002	159.3802	232.1197	323.0716	394.6686	496.8733	219.1187	275.1111	345.3221
BARLEY	250	66.378	91.2914	136.5331	198.4244	325.1975	400.4625	160.181	205.9434	233.3933
BARLEY	500	75.057	99.7028	138.1832	304.5911	364.1937	368.7941	194.9004	358.4717	567.8317
BARLEY	1000	86.012	100.4193	116.8377	326.8585	370.2588	404.4023	255.2328	396.0264	505.5334
BARLEY	2000	96.159	103.1696	116.3037	478.3594	525.7576	549.7203	368.8762	810.5905	1.48 × 10^3^
WIN95PTS	250	649.75	1.10 × 10^3^	1.52 × 10^3^	1.81 × 10^3^	4.44 × 10^3^	7.16 × 10^3^	1.60 × 10^4^	2.06 × 10^4^	2.31 × 10^4^
WIN95PTS	500	555.26	727.4609	819.2716	2.33 × 10^3^	4.76 × 10^3^	6.67 × 10^3^	6.23 × 10^3^	1.83 × 10^4^	2.48 × 10^4^
WIN95PTS	1000	693.01	746.7864	827.4684	2.73 × 10^3^	4.38 × 10^3^	6.00 × 10^3^	2.01 × 10^4^	2.57 × 10^4^	3.19 × 10^4^
WIN95PTS	2000	715.72	1.43 × 10^3^	1.85 × 10^3^	2.25 × 10^3^	7.04 × 10^3^	1.50 × 10^4^	2.13 × 10^4^	3.44 × 10^4^	4.98 × 10^4^
PIGS	250	1.87 × 10^4^	2.52× 10^4^	3.96 × 10^4^	2.60 × 10^5^	3.89 × 10^5^	4.85 × 10^5^	1.45 × 10^5^	2.48 × 10^5^	3.77 × 10^5^
PIGS	500	5.35 × 10^4^	6.84 × 10^4^	8.53 × 10^4^	3.09 × 10^5^	4.03 × 10^5^	5.24 × 10^5^	1.58 × 10^5^	2.56 × 10^5^	3.03 × 10^5^
PIGS	1000	7.12 × 10^4^	8.64 × 10^4^	9.71 × 10^4^	2.92 × 10^5^	4.14 × 10^5^	4.59 × 10^5^	1.85 × 10^5^	2.70 × 10^5^	3.57 × 10^5^
PIGS	2000	6.17 × 10^4^	9.00 × 10^4^	1.09 × 10^5^	4.26 × 10^5^	5.26 × 10^5^	7.05 × 10^5^	1.98 × 10^5^	2.74 × 10^5^	3.33 × 10^5^
MINUN	250	1.98 × 10^5^	2.70 × 10^5^	4.33 × 10^5^	1.71 × 10^6^	2.70 × 10^6^	3.46 × 10^6^	4.54 × 10^5^	7.74 × 10^5^	1.09 × 10^6^
MINUN	500	3.10 × 10^5^	4.05 × 10^5^	4.98 × 10^5^	2.52 × 10^6^	3.24 × 10^6^	4.17 × 10^6^	5.45 × 10^5^	9.00 × 10^5^	1.07 × 10^6^
MINUN	1000	3.39 × 10^5^	4.14 × 10^5^	4.72 × 10^5^	2.43 × 10^6^	3.41 × 10^6^	3.88 × 10^6^	8.19 × 10^5^	1.17 × 10^6^	1.57 × 10^6^
MINUN	2000	2.93 × 10^5^	4.23 × 10^5^	5.06 × 10^5^	2.93 × 10^6^	3.67 × 10^6^	4.99 × 10^6^	9.81 × 10^5^	1.35 × 10^6^	1.65 × 10^6^
